# Impact of Overhydration on Left Ventricular Hypertrophy in Patients With Chronic Kidney Disease

**DOI:** 10.3389/fnut.2022.761848

**Published:** 2022-02-25

**Authors:** Lianqin Sun, Qing Li, Zhiying Sun, Suyan Duan, Guangyan Nie, Jiaxin Dong, Chengning Zhang, Ming Zeng, Bin Sun, Yanggang Yuan, Ningning Wang, Huijuan Mao, Changying Xing, Bo Zhang

**Affiliations:** Department of Nephrology, The First Affiliated Hospital of Nanjing Medical University, Jiangsu Province Hospital, Nanjing Medical University, Nanjing, China

**Keywords:** overhydration, left ventricular mass index, left ventricular hypertrophy, chronic kidney disease, odds ratio

## Abstract

**Objective:**

Volume overload is a frequent feature related to left ventricular hypertrophy (LVH) in dialysis patients, but its influence on patients with chronic kidney disease (CKD) not on dialysis has not been accurately uncovered. This article was to examine the relationship between overhydration (OH) and LVH in patients with CKD not yet on dialysis.

**Methods:**

A total of 302 patients with CKD stages 1–4 were included. Participants were divided into different subgroups according to occurring LVH or not, and OH tertiles. Clinical and laboratory parameters were compared among groups. Spearman correlation analyses were adopted to explore the relationships of echocardiographic findings with the clinical and laboratory characteristics. Binary logistic regression models were performed to estimate the odds ratios (ORs) for the associations between OH and LVH. Restricted cubic splines were implemented to assess the possible non-linear relationship between OH and LVH. LVH was defined as left ventricular mass index (LVMI) >115 g/m^2^ in men and >95 g/m^2^ in women.

**Results:**

Of the enrolled patients with CKD, the mean age was 45.03 ± 15.14 years old, 165 (54.6%) cases were men, and 65 (21.5%) cases had LVH. Spearman correlation analyses revealed that OH was positively correlated with LVMI (*r* = 0.263, *P* < 0.001). After adjustment for age, gender, diabetes, body mass index (BMI), systolic blood pressure (SBP), hemoglobin, serum albumin, estimated glomerular filtration rate (eGFR), and logarithmic transformation of urinary sodium and urinary protein, multivariate logistic regression analyses demonstrated that both the middle and highest tertile of OH was associated with increased odds of LVH [OR: 3.082 (1.170–8.114), *P* = 0.023; OR: 4.481 (1.332–15.078), *P* = 0.015, respectively], in comparison to the lowest tierce. Restricted cubic spline analyses were employed to investigate the relationship between OH and LVH, which unfolded a significant non-linear association (*P* for non-linear = 0.0363). Furthermore, patients were divided into two groups according to CKD stages. The multivariate logistic regression analyses uncovered that increased odds of LVH were observed in the middle and the highest tertile of OH [OR: 3.908 (0.975–15.670), *P* = 0.054; OR: 6.347 (1.257–32.054), *P* = 0.025, respectively] in patients with stages 1–2.

**Conclusion:**

These findings suggest that a higher level of OH was associated with a higher occurrence of LVH in patients with CKD not on dialysis, especially in patients with CKD stages 1–2.

## Introduction

As chronic kidney disease (CKD) progresses, the prevalence of cardiovascular disease (CVD) and cardiac death is strikingly rising (1). Increasing evidence has revealed that CVD instead of end-stage renal disease (ESRD) is a real burden in patients with mild-to-moderate renal injury (2–4). Of all the cardiac problems in patients with CKD, left ventricular hypertrophy (LVH), histologically characterized by myocardial fibrosis, is the most common structural impairment (5). LVH not only accelerates renal dysfunction but also elevates the proportion of sudden cardiac death in patients with CKD (6–8). For decades, approaches to predict LVH have been constantly studied and mostly rely upon the clinical factors containing blood pressure, BMI, estimated glomerular filtration rate, serum phosphate, and fibroblast growth factor 23 (9–13). However, it is indispensable to discover novel predictors for LVH in patients with CKD, which is beneficial to more effective therapeutic interventions.

Volume expansion is a common complication of CKD (14) and is frequently related to inflammation, CKD progression, and mortality (15–17). Therefore, exact assessment and management of fluid status are of paramount importance in these patients. In comparison with conventional tools to evaluate fluid volume, bioelectrical impedance analysis (BIA) has been a simple, non-invasive, and high-efficient means, and is widely used in patients with CKD (18). Until recently, overhydration (OH), generated from a three-compartment model, has replaced the ratios of extracellular water (ECW)/total body water (TBW) and ECW/intracellular water (ICW) as the representative of evaluating volume status (19). Previous studies have reported the predictive impacts of OH on LVH in patients with CKD stage 5 (20, 21). However, it has not been adequately illustrated about these parameters in patients with an estimated glomerular filtration rate (eGFR) of more than 15 ml/min/1.73 m^2^. With this aim in mind, this study investigated the association of OH with clinical features and LVH in patients with CKD not yet on dialysis.

## Materials and Methods

### Subjects

**Figure 1:**
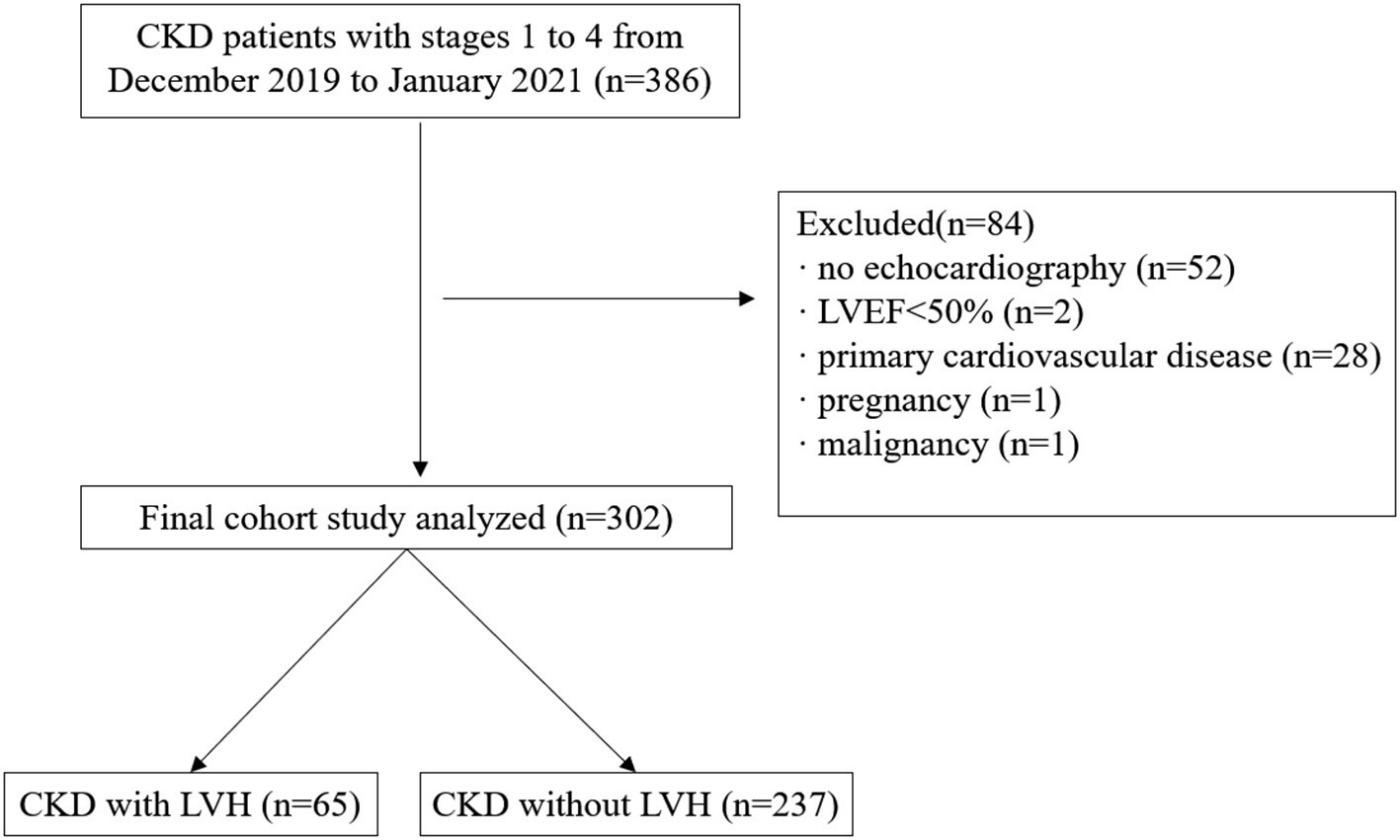
Flowchart of study participants. CKD, chronic kidney disease; LVEF, left ventricular ejection fraction; LVH, left ventricular hypertrophy.

A total of 386 patients with CKD with stages 1–4 from December 2019 to January 2021 were retrospectively reviewed and they were treated at the department of nephrology, the First Affiliated Hospital of Nanjing Medical University. The inclusion criteria were patients diagnosed with CKD, whose eGFR >15 ml/min/1.73 m^2^. CKD is defined as abnormalities of kidney structure or function, present for >3 months in accordance with the guidelines of the Kidney Disease: Improving Global Outcomes (KDIGO) Clinical Practice (22). Exclusion criteria were as follows: (1) lack of body composition index and echocardiography information; (2) implantation of a cardiac pacemaker, defibrillator, or metallic objects; (3) the amputation of any extremity; (4) clinical state affecting body composition, such as liver cirrhosis, active infectious disease or acute cardiovascular events within 3 months before screening for inclusion; (5) left ventricular ejection fraction (LVEF) <50%; (6) comorbid cardiovascular disease, such as coronary artery disease, atrial fibrillation, valvular heart disease or primary cardiomyopathy; (7) pregnancy and malignancy. Ultimately, 302 participants were included (Figure 1).

### Clinical and Laboratory Measurements

The patient's clinical characteristics containing age, gender, BMI, systolic blood pressure (SBP), diastolic blood pressure (DBP), mean arterial pressure (MAP), history of hypertension and diabetes, and current medication were recorded at the enrollment. The plasma levels of creatinine, albumin, triglyceride (TG), total cholesterol (TC), low-density lipoprotein cholesterol (LDL-C), high-density lipoprotein cholesterol (HDL-C), hemoglobin, and C-reactive protein (CRP) were measured using blood samples taken from patients who were under fasting condition. In order to assess urinary protein, sodium, and potassium, patients were required to simultaneously gather 24-h urine specimens. eGFR was calculated by the Chronic Kidney Disease Epidemiology Collaboration (CKD-EPI)^2009^ equation (23).

### Body Composition Measurements

Assessment of volume status was performed on a body composition monitor (BCM) (Fresenius Medical Care, Bad Homburg, Germany) by the same experienced nurse according to the instrument instructions. The patient's clinical parameters containing age, gender, height, and weight were inputted into the device. Electrodes were placed on the hand and foot of patients' non-dominant side, and then electrical responses were collected every 50 discrete frequencies from 5 to 1,000 kHZ. Given the measured impedance information, TBW, ECW, and intracellular water (ICW) were calculated by the equations proposed by Moissl et al. (24). Over the next few years, Chamney et al. (19) proposed a three-compartment model to describe OH in absolute liters. The model consists of lean tissue mass, adipose tissue mass, and OH. OH is calculated by subtracting water in the tissue measured by physiologic models under normal status from ECW present in tissue that was actually measured. Fluid overload (FO) was defined as an absolute OH ≥1.1 L in this study (14).

### Echocardiographic Measurements

Echocardiography was performed using an ultrasound machine (Vivid E9; GE Vingemed Ultrasound AS, Horten, Norway) with a 2.5-MHz transducer by a single experienced cardiologist who was completely blinded to the patient information. M-mode and two-dimensional measurements were used to perform cardiac chamber quantification in accordance with the guidelines of the American Society of Echocardiography (25). Thereafter, the dimensions of the interventricular septal thickness (IVST), left ventricular end-diastolic dimension (LVDd), left ventricular posterior wall thickness (PWT), left atrial dimension (LAD), and LVEF using the biplane Simpson's method were measured. IVST and PWT were measured at end-diastole. At the level of the mitral valve tips during diastole, pulse-wave Doppler was employed to observe transmitral early diastolic velocity (E), peak velocity flow in the late diastole caused by atrial contraction (A), and their deceleration time in the apical four-chamber view. Meanwhile, early diastolic mitral annular tissue velocity (e'), the average of septal and lateral mitral annular velocities, was calculated as well as E/e'. The following equations were performed according to the American Society of Echocardiography guidelines (25). Left ventricular mass = 0.8 × 1.04 × [(IVST + LVDd + PWT)^3^ – LVDd^3^] + 0.6 (g). Body surface area (BSA) = (0.007184 × weight^0.425^ × height^0.725^) m^2^. Left ventricular mass index (LVMI) = LVM/BSA (g/m^2^). Relative wall thickness (RWT) = 2 × PWT/LVDd. The left ventricular hypertrophy (LVH) was defined as an LVMI > 115 g/m^2^ in men and > 95 g/m^2^ in women (25).

### Statistical Analysis

Participants were categorized into subgroups according to occurring LVH or not, and OH tertiles. Data were presented as mean ± SD, median, and interquartile range or percentage as appropriate. Comparisons between groups were performed using Student's *t*-test or ANOVA, Mann–Whitney test, Kruskal–Wallis test, or chi-squared test as appropriate. Spearman correlation analyses were adopted to explore the relationships of echocardiographic findings with clinical and laboratory parameters. The ORs for the associations between OH and LVH were evaluated by binary logistic regression models. Crucial covariates for LVH, including age, gender, diabetes, BMI, SBP, hemoglobin, serum albumin, eGFR, urinary sodium excretion, and urinary protein excretion, were selected based on prior knowledge and the findings of univariable analyses were adjusted in multivariable analyses. Possible non-linear relationships between OH and LVH were examined with restricted cubic splines using the same multivariable model (26). *P* < 0.05 was considered statistically significant. All the statistics were done in IBM SPSS version 20.0 and R version 4.1.0.

## Results

### Baseline Clinical and Laboratory Characteristics of Patients With CKD

In total, 302 patients with CKD stages 1–4 were recruited in this study (Table 1). Their mean age was 45.03 ± 15.14 years old, and 54.6% of patients were men. Patients with CKD stages 1, 2, 3, and 4 accounted for 53.0, 22.8, 17.9, and 6.3% of the enrolled patients, respectively. LVH was present in 21.5% of all the enrolled patients. Suffering from LVH was 40 (17.5%) cases in CKD stages 1–2 but 25 (34.2%) cases in CKD stages 3–4. There was a significant increase in the prevalence of LVH in patients with CKD 3–4 as opposed to CKD 1–2 (*P* = 0.002). A total of 140 (46.4%) cases had hypertension, and 51 (16.9%) cases had diabetes. Among all the enrolled participants, the median OH, LVMI, urinary protein excretion, and eGFR were 0.50 L [interquartile range (IQR) −0.50–1.93], 87.91 g/m^2^ (IQR 74.77–99.79), 1.14 g/d (IQR 0.38–3.91), and 92.95 ml/min/1.73 m^2^ (IQR 61.24–111.97), respectively. Next, patients were categorized into two groups according to the occurrence of LVH. Compared with the patients without LVH, significantly higher levels of age, SBP, MAP, LAD, LVDd, LVMI, RWT, E/e' ratio, urinary protein excretion, and OH were observed in patients with LVH, and the lower levels of LVEF, E/A ratio, eGFR, serum albumin, hemoglobin, and ICW. Moreover, female sex, hypertension, diabetes, FO, and the use of medications containing calcium-channel blocker (CCB) and β-blocker were more prevalent among patients with LVH (all *P* < 0.05, Table 1).

**Table 1:** Comparisons of clinical, laboratory, and echocardiographic parameters and volume status between the non-LVH and LVH groups in patients with CKD.

	**Total (*n* = 302)**	**Non-LVH (*n* = 237)**	**LVH (*n* = 65)**	***P*-value**
Age (years)	45.03 ± 15.14	42.69 ± 14.66	53.58 ± 13.83	**<0.001**
Sex (male/female)	165/137	140/97	25/40	**0.003**
BMI (kg/m^2^)	24.61 ± 3.70	24.51 ± 3.74	24.97 ± 3.58	0.377
SBP (mmHg)	130.59 ± 18.24	127.98 ± 16.77	140.09 ± 20.27	**<0.001**
DBP (mmHg)	81.85 ± 12.47	81.25 ± 12.60	84.02 ± 11.85	0.114
MAP (mmHg)	98.09 ± 13.26	96.83 ± 13.11	102.71 ± 12.86	**0.001**
Hypertension (%)	140 (46.4)	92 (38.8)	48 (73.8)	**<0.001**
Diabetes (%)	51 (16.9)	29 (12.2)	22 (33.8)	**<0.001**
LAD (cm)	3.30 (3.00, 3.70)	3.20 (2.90, 3.50)	3.70 (3.20, 4.00)	**<0.001**
LVDd (cm)	4.60 (4.30, 4.90)	4.50 (4.20, 4.80)	4.90 (4.70, 5.10)	**<0.001**
LVMI (g/m^2^)	87.91 (74.77, 99.79)	83.34 (71.49, 91.21)	113.43 (102.53, 127.88)	**<0.001**
RWT	0.42 (0.39, 0.44)	0.42 (0.38, 0.44)	0.43 (0.41, 0.46)	**0.004**
LVEF (%)	64.20 (62.40, 65.80)	64.40 (62.70, 65.90)	63.70 (61.80, 65.40)	**0.007**
E/e' ratio	7.05 (6.00, 9.10)	6.80 (5.80, 8.25)	9.50 (7.35, 11.55)	**<0.001**
E/A ratio	0.90 (0.70, 1.30)	1.00 (0.80, 1.30)	0.80 (0.70, 1.20)	**0.005**
eGFR (ml/min/1.73 m^2^)	92.95 (61.24, 111.97)	96.12 (69.84, 114.18)	77.98 (41.05, 103.25)	**0.001**
Scr (μmol/L)	79.15 (60.15, 112.95)	79.00 (60.35, 107.15)	83.10 (58.25, 134.50)	0.346
Serum albumin (g/L)	35.45 (26.40, 39.60)	36.50 (27.10, 39.90)	30.10 (25.35, 37.15)	**0.010**
TG (mmol/L)	1.59 (1.09, 2.30)	1.57 (1.08, 2.31)	1.66 (1.14, 2.31)	0.482
TC (mmol/L)	5.10 (4.20, 6.26)	4.99 (4.20, 6.10)	5.23 (4.28, 6.63)	0.362
LDL-C (mmol/L)	3.03 (2.42, 3.77)	2.96 (2.43, 3.77)	3.18 (2.40, 3.93)	0.557
HDL-C (mmol/L)	1.14 (0.96, 1.38)	1.14 (0.96, 1.40)	1.14 (0.94, 1.35)	0.659
Hemoglobin (g/L)	128.43 ± 21.78	131.77 ± 20.92	116.28 ± 20.64	**<0.001**
CRP (mg/L)	1.87 (1.25, 3.18)	1.80 (1.23, 3.04)	2.00 (1.52, 3.62)	0.073
Urinary protein (g/d)	1.14 (0.38, 3.91)	0.92 (0.34, 3.01)	2.27 (0.86, 5.65)	**0.001**
Urinary sodium (mmol/d)	125.75 (86.85, 166.38)	120.60 (86.30, 166.65)	136.20 (89.55, 167.65)	0.324
Urinary potassium (mmol/d)	30.20 (23.15, 38.20)	30.30 (22.90, 38.00)	29.60 (23.60, 38.50)	0.758
OH (L)	0.50 (-0.50, 1.93)	0.20 (-0.65, 1.55)	1.30 (0.25, 3.60)	**<0.001**
ECW (L)	15.55 (13.30, 18.70)	15.50 (13.30, 18.50)	15.90 (13.45, 19.65)	0.422
ICW (L)	19.20 (16.00, 22.65)	19.70 (16.35, 22.95)	17.40 (14.95, 20.65)	**0.005**
TBW (L)	34.90 (29.40, 41.70)	35.30 (29.50, 41.90)	34.00 (28.20, 40.20)	0.244
FO (%)	110 (36.4)	74 (31.2)	36 (55.4)	**<0.001**
ACEI/ARB (%)	125 (41.4)	94 (39.7)	31 (47.7)	0.244
CCB (%)	94 (31.1)	57 (24.1)	37 (56.9)	**<0.001**
β-blocker (%)	25 (8.3)	14 (5.9)	11 (16.9)	**0.004**
Diuretic (%)	21 (7.0)	17 (7.2)	4 (6.2)	0.991

*CKD, chronic kidney disease; LVH, left ventricular hypertrophy; BMI, body mass index; SBP, systolic blood pressure; DBP, diastolic blood pressure; MAP, mean arterial pressure; eGFR, estimated glomerular filtration rate; Scr, serum creatinine; HDL-C, high-density lipoprotein cholesterol; LDL-C, low-density lipoprotein cholesterol; TG, triglyceride; TC, total cholesterol; CRP c,-reactive protein; LAD, left atrial dimension; LVDd, left ventricular end-diastolic dimension; LVMI, left ventricular mass index; RWT, relative wall thickness; LVEF, left ventricular ejection fraction; OH, overhydration; ECW, extracellular water; ICW, intracellular water; TBW, total body water; FO fluid overload; ACEI/ARB, angiotensin converting enzyme inhibitor/angiotensin receptor blockers; CCB, calcium-channel blocker*.

*Data were presented as the mean ± SD, the median with interquartile range or counts, and percentages. A two-tailed p < 0.05 was considered statistically significant. The bold values mean that P < 0.05*.

### Comparisons of Clinical and Laboratory Parameters According to OH Tertiles

On account of the finding that OH level obviously distinguished between patients with and without the occurrence of LVH, we further divided patients into three groups according to OH tertiles, and then compared the clinical and laboratory features among them (Table 2). Patients with the highest tertile of OH had the highest levels of age, SBP, MAP, LAD, LVDd, LVMI, E/e' ratio, TG, TC, LDL-C, HDL-C, urinary protein excretion, urinary sodium excretion, ECW, and TBW, along with the lowest levels of serum albumin and hemoglobin (all *P* < 0.05). While the levels of age, SBP, MAP, LAD, LVDd, LVMI, E/e' ratio, TG, TC, LDL-C, HDL-C, urinary protein excretion, urinary sodium excretion, ECW, and TBW were the lowest in the lowest tierce, which had the highest serum albumin and hemoglobin levels (all *P* < 0.05). In addition, the incidence of the male sex, diabetes, LVH, and the usage rate of CCB and diuretics were the most prevalent in patients with the highest OH tertile (all *P* < 0.05). However, no significant differences were observed in BMI, eGFR, serum creatinine, CRP, and urinary potassium excretion. Also, the incidence of hypertension and usage rate of β-blocker and angiotensin-converting enzyme inhibitors/angiotensin receptor blockers (ACEI/ARB) did not differ among the three groups according to OH tertiles.

**Table 2:** Comparisons of clinical, laboratory, and echocardiographic parameters and volume status according to OH tertiles in patients with CKD.

**Parameter**	**OH (L)**			***P*-value**
	**Tertile 1 (** ≤−**0.1)**	**Tertile 2 (–0.1–1.2)**	**Tertile 3 (>1.2)**	
*n*	105	99	98	
Age (years)	40.72 ± 12.87	44.33 ± 14.83	50.36 ± 16.20	**<0.001**
Sex (male/female)	57/48	45/54	63/35	**0.029**
BMI (kg/m^2^)	24.65 ± 3.99	24.36 ± 3.51	24.81 ± 3.60	0.693
SBP (mmHg)	124.81 ± 16.14	129.98 ± 16.06	137.39 ± 20.23	**<0.001**
DBP (mmHg)	80.10 ± 13.32	82.18 ± 12.02	83.38 ± 11.87	0.166
MAP (mmHg)	95.01 ± 13.44	98.11 ± 12.48	101.38 ± 13.15	**0.003**
Hypertension (%)	43 (41.0)	44 (44.4)	53 (54.1)	0.155
Diabetes (%)	11 (10.5)	10 (10.1)	30 (30.6)	**<0.001**
LAD (cm)	3.20 (2.90, 3.50)	3.20 (2.90, 3.40)	3.55 (3.20, 3.90)	**<0.001**
LVDd (cm)	4.50 (4.30, 4.80)	4.70 (4.20, 4.90)	4.75 (4.50, 5.00)	**<0.001**
LVMI (g/m^2^)	83.54 (72.57, 92.07)	87.86 (71.55, 99.02)	93.47 (78.44, 110.84)	**<0.001**
RWT	0.42 (0.39, 0.44)	0.42 (0.39, 0.44)	0.42 (0.38, 0.44)	0.977
LVEF (%)	64.40 (63.00, 65.70)	64.40 (63.00, 65.80)	63.50 (61.90, 65.65)	0.079
E/e' ratio	6.60 (5.80, 7.60)	7.10 (6.00, 9.00)	8.40 (6.38, 10.50)	**<0.001**
E/A ratio	1.00 (0.80, 1.30)	0.90 (0.80, 1.30)	0.90 (0.70, 1.20)	0.248
LVH (%)	8 (7.6)	24 (24.2)	33 (33.7)	**<0.001**
eGFR (ml/min/1.73 m^2^)	98.81 (57.30, 116.30)	93.88 (69.39, 111.38)	86.59 (53.12, 107.41)	0.118
Scr (μmol/L)	79.30 (63.30, 116.80)	74.50 (57.30, 101.50)	82.65 (62.00, 119.75)	0.156
Serum albumin (g/L)	38.60 (36.25, 41.85)	36.60 (30.70, 39.60)	22.40 (18.28, 31.20)	**<0.001**
TG (mmol/L)	1.42 (1.00, 2.01)	1.56 (1.02, 2.27)	1.88 (1.27, 2.50)	**0.007**
TC (mmol/L)	4.57 (3.88, 5.38)	4.78 (4.04, 5.72)	6.16 (5.12, 7.85)	**<0.001**
LDL-C (mmol/L)	2.78 (2.21, 3.35)	2.89 (2.40, 3.50)	3.65 (2.89, 4.76)	**<0.001**
HDL-C (mmol/L)	1.06 (0.94, 1.27)	1.09 (0.95, 1.34)	1.27 (1.07, 1.52)	**<0.001**
Hemoglobin (g/L)	136.80 ± 19.54	128.21 ± 18.81	119.69 ± 23.50	**<0.001**
CRP (mg/L)	2.05 (1.47, 3.59)	1.65 (1.16, 2.73)	1.94 (1.30, 3.06)	0.061
Urinary protein (g/d)	0.59 (0.30, 1.05)	0.91 (0.25, 2.76)	5.45 (1.90, 8.63)	**<0.001**
Urinary sodium (mmol/d)	109.90 (77.10, 151.05)	122.00 (88.90, 165.90)	140.55 (108.40, 192.65)	**0.002**
Urinary potassium (mmol/d)	30.60 (22.80, 39.10)	28.00 (22.40, 35.10)	30.95 (24.60, 41.38)	0.105
OH (L)	−0.80 (−1.30, −0.45)	0.50 (0.20, 0.90)	3.30 (2.00, 5.65)	**<0.001**
ECW (L)	14.10 (12.35, 16.10)	15.00 (13.10, 17.60)	19.10 (15.93, 22.05)	**<0.001**
ICW (L)	19.50 (16.30, 22.40)	18.70 (15.50, 22.50)	19.65 (16.00, 23.10)	0.547
TBW (L)	33.70 (28.70, 38.85)	33.90 (28.20, 39.70)	39.00 (33.30, 45.83)	**<0.001**
ACEI/ARB (%)	46 (43.8)	33 (33.3)	46 (46.9)	0.126
CCB (%)	25 (23.8)	30 (30.3)	39 (39.8)	**0.048**
β-blocker (%)	4 (3.8)	9 (9.1)	12 (12.2)	0.087
Diuretic (%)	2 (1.9)	5 (5.1)	14 (14.3)	**0.002**

*CKD, chronic kidney disease; BMI, body mass index; SBP, systolic blood pressure; DBP, diastolic blood pressure; MAP, mean arterial pressure; eGFR, estimated glomerular filtration rate; Scr, serum creatinine; HDL-C, high-density lipoprotein cholesterol; LDL-C, low-density lipoprotein cholesterol; TG, triglyceride; TC, total cholesterol; CRP c,-reactive protein; LAD, left atrial dimension; LVDd, left ventricular end-diastolic dimension; LVMI, left ventricular mass index; RWT, relative wall thickness; LVEF, left ventricular ejection fraction; OH, overhydration; ECW, extracellular water; ICW, intracellular water; TBW, total body water; ACEI/ARB, angiotensin converting enzyme inhibitor/ angiotensin receptor blockers; CCB, calcium-channel blocker*.

*Data were presented as the mean ±SD, the median with interquartile range or counts, and percentages. A two-tailed p < 0.05 was considered statistically significant. The bold values mean that P < 0.05*.

### Correlations Between Echocardiographic Findings and Clinical and Laboratory Parameters

**Figure 2:**
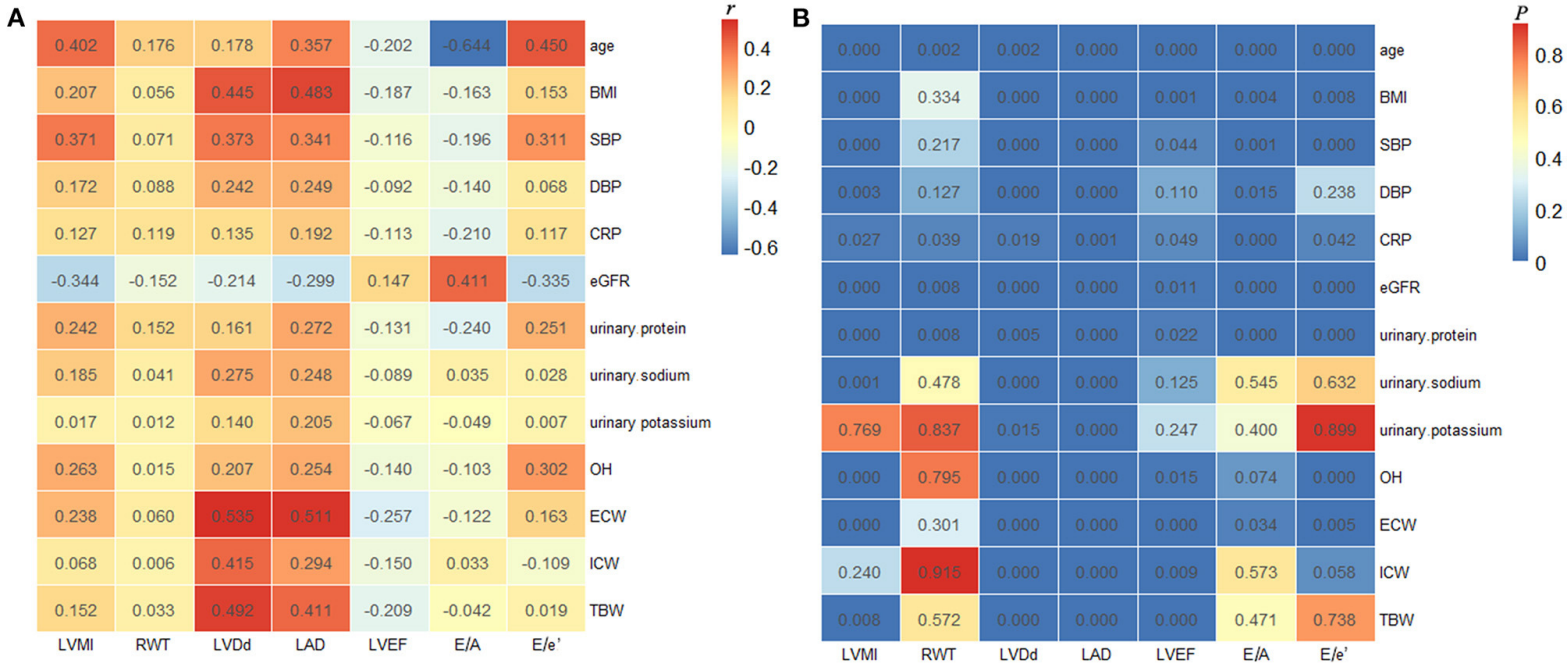
Correlations between echocardiographic findings and clinical and laboratory parameters. The correlation coefficients **(A)** and *P-*values **(B)** of Spearman correlation analyses in all the enrolled patients. LVMI, left ventricular mass index; RWT, relative wall thickness; LVDd, left ventricular end-diastolic dimension; LAD, left atrial dimension; LVEF, left ventricular ejection fraction; BMI, body mass index; SBP, systolic blood pressure; DBP, diastolic blood pressure; CRP, C-reactive protein; eGFR, estimated glomerular filtration rate; OH, overhydration; ECW, extracellular water; ICW, intracellular water; TBW, total body water.

As shown in Figure 2, Spearman correlation analyses indicated that LVMI positively correlated with OH (*r* = 0.263, *P* < 0.001), ECW (*r* = 0.238, *P* < 0.001), TBW (*r* = 0.152, *P* = 0.008), urinary sodium excretion (*r* = 0.185, *P* = 0.001), urinary protein excretion (*r* = 0.242, *P* < 0.001), CRP (*r* = 0.127, *P* = 0.027), age (*r* = 0.402, *P* < 0.001), BMI (*r* = 0.207, *P* < 0.001), SBP (*r* = 0.371, *P* < 0.001), and DBP (*r* = 0.172, *P* = 0.003), whereas it was negatively correlated with eGFR (*r* = −0.344, *P* < 0.001). Besides, OH was positively correlated with LVDd (*r* = 0.207, *P* < 0.001), LAD (*r* = 0.254, *P* < 0.001), and E/e' ratio (*r* = 0.302, *P* < 0.001), it was negatively correlated with LVEF (*r* = −0.140, *P* = 0.015), nevertheless. However, the correlations of LVMI with urinary potassium excretion and ICW, and the correlations of OH with RWT and E/A ratio showed no significant differences.

### Overhydration and Left Ventricular Hypertrophy

**Figure 3:**
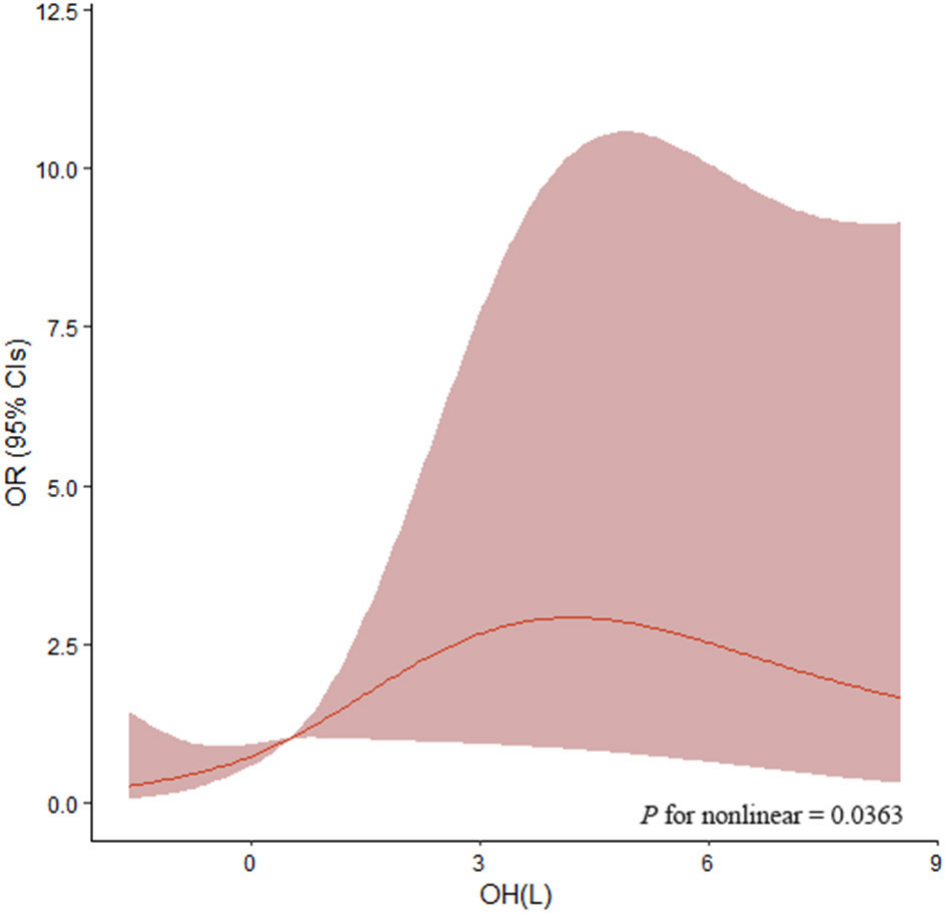
Association of OH with OR of LVH by restricted cubic splines. Restricted cubic splines were plotted using four default knots at the 20th, 40th, 60th, and 80th percentiles. ORs were adjusted for age, gender, diabetes, BMI, SBP, hemoglobin, serum albumin, eGFR, and logarithmic transformation of urinary sodium and urinary protein. The *P*-value for non-linear association was 0.0363. OH, overhydration; OR, odds ratio; BMI, body mass index; SBP, systolic blood pressure; eGFR, estimated glomerular filtration rate.

As shown in Table 3, the ORs for the associations between OH and LVH were determined by binary logistic regression models. In unadjusted models, the highest tertile [OR: 6.156 (2.674–14.171), *P* < 0.001] and middle tertile [OR: 3.880 (1.650–9.124), *P* = 0.002] of OH were significantly associated with increased odds of LVH in comparison to the lowest tertile. Also, age, gender, diabetes, SBP, hemoglobin, eGFR, and logarithmic transformation of urinary protein excretion were associated with LVH. After adjustment for age, gender, diabetes, BMI, SBP, hemoglobin, serum albumin, eGFR, and logarithmic transformation of urinary sodium and urinary protein in the multivariate logistic regression analyses, increased odds of LVH were also observed in the middle and highest tertiles of OH [OR: 3.082 (1.170–8.114), *P* = 0.023; OR: 4.481 (1.332–15.078), *P* = 0.015, respectively]. Furthermore, the same multivariable-adjusted restricted cubic spline analyses confirmed this finding, which verified a significant non-linear association between OH and LVH (*P* for non-linear = 0.0363, Figure 3). In addition, the multivariate logistic regression analysis demonstrated that age [OR: 1.037 (1.010–1.064), *P* = 0.006], gender [OR: 3.412 (1.472–7.905), *P* = 0.004], and SBP [OR: 1.024 (1.003–1.046), *P* = 0.027] were independently associated with LVH. Nevertheless, other clinical and laboratory parameters containing diabetes, BMI, hemoglobin, serum albumin, eGFR, and logarithmic transformation of urinary sodium excretion and urinary protein excretion did not relate to LVH.

**Table 3:** Univariate and multivariate logistic regression for the association between OH and LVH in all the enrolled patients with CKD.

**Variables**	**Univariate**		**Multivariate**	
	**OR (95%CI)**	***P*-value**	**OR (95%CI)**	***P*-value**
OH (L)
Tertile 1 (≤-0.1)	1 [reference]		1 [reference]	
Tertile 2 (−0.1–1.2)	3.880 (1.650, 9.124)	0.002	3.082 (1.170, 8.114)	0.023
Tertile 3 (>1.2)	6.156 (2.674, 14.171)	<0.001	4.481 (1.332, 15.078)	0.015
*P*-value for trend	<0.001		0.015	
Age (years)	1.053 (1.031, 1.075)	<0.001	1.037 (1.010, 1.064)	0.006
Gender (male vs. female)	2.309 (1.315, 4.054)	0.004	3.412 (1.472, 7.905)	0.004
Diabetes (no vs. yes)	3.670 (1.927, 6.988)	<0.001	1.797 (0.768, 4.205)	0.176
BMI (kg/m^2^)	1.034 (0.961, 1.112)	0.376	1.064 (0.958, 1.182)	0.245
SBP (mmHg)	1.038 (1.021, 1.054)	<0.001	1.024 (1.003, 1.046)	0.027
Hemoglobin (g/L)	0.966 (0.952, 0.979)	<0.001	0.981 (0.961, 1.002)	0.077
Serum albumin (g/L)	0.970 (0.941, 1.000)	0.052	1.060 (0.986, 1.140)	0.114
eGFR (ml/min/1.73 m^2^)	0.985 (0.976, 0.993)	<0.001	0.999 (0.985, 1.013)	0.873
Log urinary sodium^* ^(mmol/d)	1.471 (0.398, 5.437)	0.563	0.857 (0.144, 5.105)	0.865
Log urinary protein^* ^(g/d)	2.149 (1.355, 3.406)	0.001	1.622 (0.629, 4.184)	0.317

*CKD, chronic kidney disease; OH, overhydration; LVH, left ventricular hypertrophy; BMI, body mass index; SBP, systolic blood pressure; eGFR, estimated glomerular filtration rate; OR, odds ratio*.

* *Urinary sodium and urinary protein were normalized by Log_10_ transformation*.

Furthermore, the patients were divided into two groups (group 1: patients with CKD stages 1–2; group 2: patients with CKD stages 3–4) according to CKD stages. In univariate logistic regression analyses, patients with higher tertile of OH showed a higher occurrence of LVH both in patients with stages 1–2 and in patients with stages 3–4. However, increased odds of LVH were observed in the middle and highest tertile of OH [OR: 3.908 (0.975–15.670), *P* = 0.054; OR: 6.347 (1.257–32.054), *P* = 0.025, respectively, *P* for trend = 0.026] only in patients with stages 1–2, with adjustment for age, gender, diabetes, BMI, SBP, hemoglobin, serum albumin, and logarithmic transformation of urinary sodium and urinary protein in the multivariate logistic regression analyses.

## Discussion

Our study demonstrated a significant association between OH and LVH in patients with non-dialysis CKD. First of all, our results verified LVMI was positively correlated with OH, ECW, TBW, urinary sodium excretion, and urinary protein excretion, whereas it was negatively correlated with eGFR. Furthermore, patients with the highest tertile of OH had the highest prevalence of LVH. Most importantly, both the middle and highest tertile of OH were associated with significantly increased odds of LVH after adjustment for important covariates, which indicated a higher level of OH was an independent determining factor for a higher occurrence of LVH in patients with CKD not on dialysis. In addition, restricted cubic spline analyses unfolded a significant non-linear association between OH and LVH, which suggested OH must be the dummy variable when taken into logistic regression analyses.

On account of reduced glomerular filtration rate, sodium retention, and proteinuria (27, 28), impaired volume homeostasis is one of the main characteristics of CKD, especially in the later stages. Numerous studies have identified a series of adverse effects of FO on patients with CKD. In patients with CKD, Braam et al. (29) pointed out extracellular volume expansion was associated with increased arterial stiffness and uncontrolled hypertension. Another finding from a prospective observational cohort study of CKD also observed a significant association between higher ECW/TBW ratio and increased 24-h SBP (30). Through our study, we used OH that a new, effective, and representative clinical index to assess volume status. Results showed that patients with a higher level of OH had higher SBP, which was consistent with the aforementioned studies. In addition, several studies reported that higher levels of BP and worse BP control statuses were verified related to LVH (31, 32). In line with this, we found patients with a higher level of SBP manifested increased LVMI. Most importantly, SBP was independently associated with LVH. These observations suggested BP might be an intermediate link between OH and LVH. However, the key finding of this study was that higher OH was related to increased odds of LVH independent of SBP, suggesting other unknown mechanisms were involved.

Accumulated evidence has confirmed inflammation and endothelial dysfunction linked to OH. Increase in inflammatory cytokines containing interleukin (IL)-8, IL-6, and tumor necrosis factor-α (TNF-α) and disorder in endothelial function markers including E-selectin, vascular adhesion molecule (VCAM)-1, and thrombomodulin were detectable in serum or peripheral blood cells in patients with CKD, with some increasing with overhydration (15, 33, 34). Moreover, inflammation is perceived to be probably linked to LVH in patients with CKD. Striking increased levels of serum macrophage migratory inflammatory factor, CRP, IL-1 receptor antagonist, IL-6, and TNF-α were linked to elevated odds of LVH (35, 36). Morphology and function of vascular smooth muscle cells could be altered by inflammation, causing increased arterial stiffness, accelerating the development of LVH. Meanwhile, the subclinical inflammation brings about adverse left ventricular geometry by changing the equilibrium, which adjusts cell growth, apoptosis, phenotype, and matrix turnover of cardiac tissue (37). In addition, Francis et al. (38) perceived inflammation increased bone-derived hormone fibroblast growth factor 23 (FGF23) production, a crucial regulator of mineral metabolism and biomarker of fibrosis, which has been verified to link with LVH in both the animal models and clinical experiment models (39, 40). In this study, CRP, one of the inflammation markers, was also observed positively correlated with LVMI, which suggested inflammation might take part in the latent mechanism of an obvious association between higher OH and a higher level of LVMI in patients with CKD.

Another important finding was that patients with a higher level of OH had higher proteinuria, which was a strong factor in the development of LVH (41). In addition, our findings were consistent with previous studies that elevated OH linked with worse urinary protein (34, 42). On the contrary, heavy proteinuria causes hypoproteinemia, which adds interstitial fluid volume and contracts intravascular volume by a diminished oncotic pressure gradient, inducing renal sodium retention by activation of the renin-angiotensin-aldosterone (43). On the other hand, in the rat's model that was subjected to unilateral nephrectomy and a high-salt diet, it was unfolded that fluid retention was associated with an increase in the renal inflammation with macrophage infiltration and tumor necrosis factor-α overexpression, and glomerular sclerosis (44). Therefore, volume overload might be involved in an aggravating renal injury, which results in massive urinary protein.

Interestingly, our data showed that urinary sodium excretion did not relate to LVH in patients with CKD. Dhingra et al. (45). also observed urinary sodium excretion measured on a spot urine sample was not linked with LVH. Nevertheless, in the study by Zhang et al. (46), the highest tertile of night/day urinary sodium excretion ratio was independently associated with LVH in Chinese patients with CKD. The discrepancy in results may be due to differences in the methods of measurement and ranges of urinary sodium excretion, study populations, and failure to explore non-linear associations (47). Also, there was no significant association between the severity of CKD and LVH. One example is the wide heterogeneity in the prevalence of LVH, which may be due to the number of patients recruited, the proportion of subjects, the presence of comorbidities, and differences in the stages of CKD. FO is associated with the incidence rate of cardiovascular disease and all-cause mortality in patients with CKD without dialysis. The difference between this study and the previously mentioned results maybe because of the differences in registration criteria and patient cohort composition. Schneider et al. (48) showed that in patients with mild-to-moderate CKD, the volume status assessed by bioelectrical impedance spectroscopy (BIS) was independent of the LV mass measured by MRI. In that study, the eGFR range of enrolled patients was 41–62 ml/min and the urinary ACR range was 8–375 mg/g. They found that increased BMI, hemoglobin, and 24-h SBP were key determinants of LVH. Regression models showed that hydration status, endothelial function, inflammation, or MBD parameters did not increase the significance of LVH models.

In addition, our study found that a significant association between OH and LVH was observed in CKD patients with stages 1–2 but not in stages 3–4. Numerous studies revealed that not only FO, but also renin-angiotensin-aldosterone system (RAAS) system activation, endothelial dysfunction, and proinflammatory factors overexpression existed in CKD with moderate-to-serious renal dysfunction, which was related to LVH (15, 34, 49–51). In that case, changes in OH alone were not enough to increase the prevalence of LVH, which might explain why OH did not associate with LVH in CKD patients with stages 3–4. In addition, the numbers with CKD stages 3–4 were much lower than stages 1–2, causing this subgroup analysis underpowered, which was also the explanation of the discrepancy in associations between OH and LVH in CKD stages 1–2 and stages 3–4. Intriguingly, findings showed that the relationship between OH and LVH was stronger where the CKD is less severe (stages 1–2), and with larger ORs. Moreover, there were still 33.2% of CKD patients with stages 1–2 suffering from overhydrated (OH ≥ 1.1 L). Compared with patients without overhydrated, urinary sodium excretion, an important means to measure dietary salt intake, was significantly increased in these patients with overhydrated, which reflected higher salt and water retention. Apart from that, an article published in JAMA pointed out higher urinary sodium excretion was associated with an increased risk of cardiovascular disease in CKD patients (52). All these phenomena suggest that volume management should be initiated even in the patients with early CKD, such as dietary restriction of salt.

This study has several limitations. First, this study was cross-sectional observational research so causation could not be inferred. Moreover, while covariates affecting LVH had been adjusted as much as we could, residual confounding was still a latent limitation. Second, this study was conducted in a single center, and the sample size was small as compared with the article published in JAMA (52). Statistical power might be reduced when we performed subgroup analysis based on eGFR due to the limited samples. In particular, the sample size of the two groups was not balanced (Table 4). More samples are required in future studies to verify the results of this association in CKD stages 1–2. Third, most patients lacked other laboratory indexes reflecting fluid status and cardiac function, such as amino-terminal pro-B-natriuretic peptide (NT-proBNP), and cardiac troponin T(cTnT), which cannot be included in the subsequent analysis. Further multiple-center and controlled prospective studies on large groups of patients are needed.

**Table 4:** Univariate and multivariate logistic regression for the association between OH and LVH in CKD patients with stages 1–2 and 3–4.

**Variables**	**Univariate**		**Multivariate^a^**	
	**OR (95%CI)**	***P*-value**	**OR (95%CI)**	***P*-value**
Stages 1–2 (*n* = 229)
OH (L)				
Tertile 1 (≤-0.1)	1 [reference]		1 [reference]	
Tertile 2 (−0.1–1.1)	6.250 (1.729, 22.597)	0.005	3.908 (0.975, 15.670)	0.054
Tertile 3 (>1.1)	10.185 (2.901, 35.763)	<0.001	6.347 (1.257, 32.054)	0.025
*P*-value for trend	<0.001		0.026	
Stages 3–4 (*n* = 73)
OH (L)				
Tertile 1 (≤-0.2)	1 [reference]		1 [reference]	
Tertile 2 (−0.2–1.7)	2.240 (0.611, 8.211)	0.224	1.611 (0.311, 8.347)	0.570
Tertile 3 (>1.7)	4.200 (1.190, 14.829)	0.026	3.394 (0.277, 41.656)	0.339
*P*-value for trend	0.025		0.348	

a *Multivariable: adjusted for age, gender, diabetes, BMI, SBP, hemoglobin, serum albumin, and logarithmic transformation of urinary sodium and urinary protein*.

*CKD, chronic kidney disease; OH, overhydration; LVH, left ventricular hypertrophy; BMI, body mass index; SBP, systolic blood pressure; OR, odds ratio*.

## Conclusion

In conclusion, this study demonstrated that a higher level of OH was associated with a higher occurrence of LVH in patients with CKD not on dialysis, especially in CKD patients with stages 1–2. This association was significant regardless of age, gender, diabetes, BMI, SBP, hemoglobin, serum albumin, eGFR, urinary sodium excretion, and urinary protein excretion.

## Data Availability Statement

The original contributions presented in the study are included in the article/Supplementary Material, further inquiries can be directed to the corresponding author/s.

## Ethics Statement

The studies involving human participants were reviewed and approved by Ethics Committee of The First Affiliated Hospital of Nanjing Medical University (approval number: No. 2018-SR-250). The patients/participants provided their written informed consent to participate in this study.

## Author Contributions

LS designed and conducted the research, and analyzed the data. QL, ZS, and SD contributed to the writing and critical review of the manuscript. GN, JD, CZ, MZ, BS, YY, and NW reviewed the manuscript. CX and HM coordinated and conceived the study and revised the manuscript. BZ is the guarantor of this study and had complete access to all the data in the study. All authors have read the final manuscript and approved the submission.

## Funding

This study was supported by grants from the Natural Science Foundation of Jiangsu Province (No. BK 20151588), the National Natural Science Foundation of China (No. 82100767), and the Natural Science Foundation of Jiangsu Province (No. BK20191075).

## Conflict of Interest

The authors declare that the research was conducted in the absence of any commercial or financial relationships that could be construed as a potential conflict of interest.

## Publisher's Note

All claims expressed in this article are solely those of the authors and do not necessarily represent those of their affiliated organizations, or those of the publisher, the editors and the reviewers. Any product that may be evaluated in this article, or claim that may be made by its manufacturer, is not guaranteed or endorsed by the publisher.
