# Supplementary material for: Impact of Overhydration on Left Ventricular Hypertrophy in Patients With Chronic Kidney Disease
Source: Front Nutr. 2022 Feb 25;9:761848. doi: 10.3389/fnut.2022.761848 (PMC8916701; doi:10.3389/fnut.2022.761848)
Supplement: Supplementary file 1 [file Table_1.docx]

Supplementary Table 1. Univariate and multivariate Logistic regression for the association between OH/ECW and LVH in all enrolled CKD patients

| Variables | Univariate | | Multivariate | |
| --- | --- | --- | --- | --- |
|  | OR (95%CI) | *P*-value | OR (95%CI) | *P*-value |
| OH/ECW |  |  |  |  |
| Tertile 1 (≤-0.008) | 1[reference] |  | 1[reference] |  |
| Tertile 2 (-0.008-0.082) | 3.623(1.541, 8.522) | 0.003 | 2.653(1.018, 6.913) | 0.046 |
| Tertile 3 (>0.082) | 5.726(2.487, 13.181) | ˂0.001 | 3.153(0.936, 10.627) | 0.064 |
| *P* value for trend | ˂0.001 |  | 0.059 |  |
| Age (years) | 1.053 (1.031, 1.075) | ˂0.001 | 1.038(1.011, 1.065) | 0.005 |
| Gender (male versus female) | 2.309 (1.315, 4.054) | 0.004 | 3.064(1.346, 6.979) | 0.008 |
| Diabetes (no versus yes) | 3.670 (1.927, 6.988) | ˂0.001 | 1.778(0.764, 4.143) | 0.182 |
| BMI (kg/m^2^) | 1.034(0.961, 1.112) | 0.376 | 1.057(0.953, 1.173) | 0.291 |
| SBP (mmHg) | 1.038(1.021, 1.054) | ˂0.001 | 1.025(1.004, 1.047) | 0.019 |
| Hemoglobin(g/L) | 0.966(0.952, 0.979) | ˂0.001 | 0.979(0.959, 1.000) | 0.048 |
| Serum albumin (g/L) | 0.970(0.941, 1.000) | 0.052 | 1.054 (0.979, 1.134) | 0.161 |
| eGFR (ml/min/1.73 m²) | 0.985(0.976, 0.993) | ˂0.001 | 1.000 (0.987, 1.014) | 0.971 |
| Log Urinary sodium* (mmol/d) | 1.471 (0.398, 5.437) | 0.563 | 0.926(0.156, 5.501) | 0.932 |
| Log Urinary protein* (g/d) | 2.149 (1.355, 3.406) | 0.001 | 1.666 (0.650, 4.272) | 0.288 |

CKD chronic kidney disease; OH overhydration; ECW extracellular water; LVH left ventricular hypertrophy; BMI body mass index; SBP systolic blood pressure; eGFR estimated glomerular filtration rate; OR odds ratio.

* Urinary sodium and urinary protein were normalized by Log_10_ transformation.
