# Supplementary material for: Impact of Overhydration on Left Ventricular Hypertrophy in Patients With Chronic Kidney Disease
Source: Front Nutr. 2022 Feb 25;9:761848. doi: 10.3389/fnut.2022.761848 (PMC8916701; doi:10.3389/fnut.2022.761848)
Supplement: Supplementary file 2 [file Presentation_1.pptx]

## Slide 1
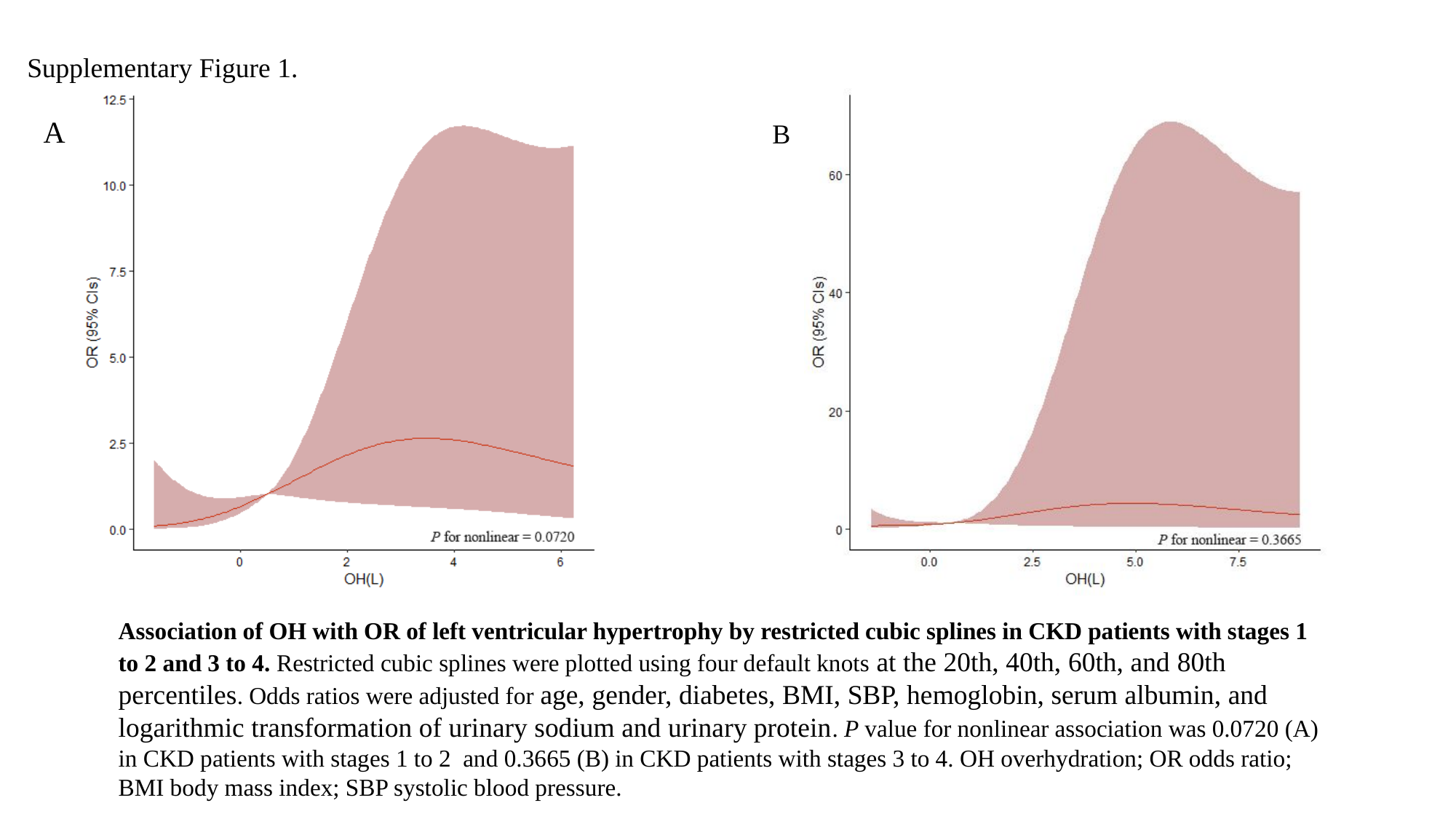

Supplementary Figure 1.
A
B
Association of OH with OR of left ventricular hypertrophy by restricted cubic splines in CKD patients with stages 1 to 2 and 3 to 4. Restricted cubic splines were plotted using four default knots at the 20th, 40th, 60th, and 80th percentiles. Odds ratios were adjusted for age, gender, diabetes, BMI, SBP, hemoglobin, serum albumin, and logarithmic transformation of urinary sodium and urinary protein. P value for nonlinear association was 0.0720 (A) in CKD patients with stages 1 to 2 and 0.3665 (B) in CKD patients with stages 3 to 4. OH overhydration; OR odds ratio; BMI body mass index; SBP systolic blood pressure.
